# *In vitro* and *in vivo* biocompatibility and bioactivity of bioceramic repair materials

**DOI:** 10.1590/1807-3107bor-2026.vol40.053

**Published:** 2026-08-07

**Authors:** Lara Cancella de ARANTES, Alexandre Henrique dos REIS-PRADO, Karina Sampaio CAIAFFA, Pedro Augusto Monteiro DELGADO, Sabrina de Castro OLIVEIRA, Francielen Oliveira FONSECA, Warley Luciano Fonseca TAVARES, Cristiane DUQUE, Luciano Tavares Angelo CINTRA, Carlos Roberto Emerenciano BUENO, Ricardo Alves de MESQUITA, Gustavo Batista de MENEZES, Antônio Paulino RIBEIRO-SOBRINHO, Francine BENETTI

**Affiliations:** (a) Universidade Federal de Minas Gerais – UFMG, School of Dentistry, Department of Restorative Dentistry, Belo Horizonte, MG, Brazil.; (b) Universidade Estadual Paulista – Unesp, School of Dentistry, Department of Pediatric Dentistry and Public Health, Araçatuba, SP, Brazil.; (c) Universidade Católica Portuguesa – UCP, Faculty of Dental Medicine, Centre for Interdisciplinary Research in Health, Viseu, Portugal.; (d) Universidade Estadual Paulista – Unesp, School of Dentistry, Department of Restorative Dentistry, Araçatuba, SP, Brazil;; (e) Universidade Federal de Minas Gerais – UFMG, School of Dentistry, Department of Dental Clinic, Pathology and Surgery, Belo Horizonte, MG, Brazil;; (f) Universidade Federal de Minas Gerais – UFMG, Institute of Biological Sciences Department of Morphology, Belo Horizonte, MG, Brazil.

**Keywords:** Biocompatible Materials, Endodontics, Models, Animal, In Vitro Technques

## Abstract

This study aimed to evaluate the *in vitro* cytotoxicity and bioactivity, and the *in vivo* biocompatibility, collagen maturation, and bioactivity, of PBS Cimmo HP and Bio-C Repair Ion+ in comparison with white MTA-Angelus. Osteoblast-like cell viability was assessed using the Alamar Blue assay, while bioactivity was assessed by alkaline phosphatase (ALP) activity and Alizarin Red staining*.* For the *in vivo* analysis, polyethylene tubes containing the materials or empty tubes (control) were implanted in the dorsum of mice. After 7 and 30 days (n = 8), the animals were euthanised and the specimens were processed for haematoxylin-eosin, Picrosirius Red, von Kossa (VK), and polarised light (PL) analyses. Data were statistically analysed (p < 0.05). Most materials extracts were cytocompatible (*P* > 0.05), except for the PBS Cimmo HP 1:4 extract at 48-h (p < 0.05). All materials showed increased ALP activity (p < 0.05). Mineralized nodules were significantly present in most material extracts (p < 0.05), except in the undiluted PBS Cimmo HP extract and in the white MTA-Angelus 1:4 extract. At 7 days, PBS Cimmo HP induced moderate-to-severe inflammation, whereas the other groups induced moderate inflammation (p > 0.05). At 30 days, all groups showed reduced inflammation (p > 0.05). Overall, the fibrous capsule was thick at 7 days and thin at 30 days. At 7 days, the control and PBS Cimmo HP groups showed a greater presence of immature collagen (p < 0.05); at 30 days, more mature collagen was observed in all groups (p > 0.05). The materials exhibited positivity for VK or PL in both periods. In conclusion, PBS Cimmo HP and Bio-C Repair Ion+ were cytocompatible and biocompatible, and demonstrated bioactivity and collagen maturation similar to those of white MTA-Angelus.

## Introduction

Biocompatibility and bioactivity are essential properties for repair materials due to their permanent contact with tissues.^1^ Mineral trioxide aggregate (MTA) is highly biocompatible and bioactive and widely used in challenging endodontic treatments such as furcation or root perforations, internal or external resorption, retrofilling in apical microsurgery, pulp capping, pulpotomy, apexification, and apexogenesis. However, MTA has some drawbacks, including difficult handling and a sandy consistency.^2^ To overcome the limitations of MTA, new repair materials have been continually developed.^3^ These new materials primarily aim to improve handling, but also to enhance formulation properties. Nevertheless, their biological behavior compared with MTA must be carefully evaluated, including cytotoxicity, bioactivity, and *in vivo* performance.

PBS Cimmo HP^4^ (MJS Ind. Materiais para Saúde LTDA, Pouso Alegre, MG) is a slow-setting bioceramic material that has shown transient cytotoxicity, induction of tumour necrosis factor (TNF)-α in human periodontal ligament stem cells (hPDLSCs),^5^ and promotion of cell proliferation even under inflammatory conditions.^6^ Bio-C Repair Ion+ (Angelus Ind. Odontológico S/A, Londrina, Brazil) is another bioceramic material available in a ready-to-use syringe. Its precursor, Bio-C Repair, showed excellent biocompatibility compared with white MTA-Angelus (Angelus Ind. Odontológico S/A),^7^ greater adhesion strength,^8^ and higher pH.^9^ Bio-C Repair Ion+ contains calcium silicate, calcium oxide, magnesium silicate, zirconium oxide, silicon dioxide, and a dispersing agent.^10^ However, its biological properties have not yet been evaluated *in vivo*, which is an essential step since compositional changes may alter its properties.^11,12^

As repair materials must also induce biomineralization-referred to as bioactivity-to promote biological sealing and prevent the infiltration of fluids and microorganisms, this study evaluated the cytotoxicity, *in vitro* bioactivity, biocompatibility, collagen maturation, and *in vivo* bioactivity of PBS Cimmo HP and Bio-C Repair Ion+, compared with white MTA-Angelus. The null hypothesis was that there would be no difference among the materials regarding cytotoxicity, *in vitro* bioactivity, biocompatibility, collagen maturation, and *in vivo* bioactivity.

## Methods

### Materials preparation

PBS Cimmo HP and white MTA-Angelus were prepared according to the manufacturer’s instructions. On a sterilized glass plate, the contents of one measuring spoon of the material were mixed with one drop of the distilled water provided with the material and spatulated for 30 seconds. Bio-C Repair Ion+ is supplied in a ready-to-use form.

### In vitro analysis

Three discs of each material (diameter: 5mm; height: 3 mm) were prepared under aseptic conditions and kept in an incubator at 37°C (6 h, 5% CO_2_). The discs were then removed from the mold and sterilized via ultraviolet irradiation (1 h). Discs from each group were placed in 2 mL of Dulbecco’s Modified Eagle’s Medium (DMEM) and incubated for 48 h to obtain the material extracts.

Osteoblastic cells (Saos-2, ATCC HTB-85) were cultivated in DMEM supplemented with 10% fetal bovine serum (FBS), penicillin (100 IU/mL), streptomycin (100 µg/mL), and 2 mmol/L glutamine. The cells were maintained in an incubator with 5% CO_2_ at 37ºC, and subcultured every 2 days. Cells were seeded (1-5 x 10^5^ cells/well) and pre-incubated for 24 h. After this period, the cells were treated with extracts of the materials in three concentrations: undiluted, 1:2, and 1:4 dilutions. Cells were exposed to the extracts for 48 h and evaluated after 24 h, 48 h, 8 days, and 14 days. Cell viability was assessed using the resazurin method (Alamar Blue; Sigma Aldrich, Burlington, USA). Absorbance was measured at 570 nm and 600 nm using a spectrophotometer. The values were converted into percentages of cell viability, considering cell growth in DMEM as 100%, and the means values were calculated for each group. Each condition was analysed in triplicate.

Alkaline phosphatase (ALP) activity was evaluated using osteogenic medium (DMEM containing 10% FBS, antibiotics, and glutamine) supplemented with 10 nmol/L of β-glycerophosphate, 50 μg/mL ascorbic acid, and 1.8 mmol/L KH_2_PO_4_. Extracts of the materials (undiluted, 1:2, and 1:4 dilutions) were added to DMEM medium (100, 50 and 25 μM) and incubated for 48 h, followed by daily replacement with osteogenic medium until completing 8 days. The ALP assay was then performed (Labtest Diagnostics S.A., Lagoa Santa, Brazil), and absorbance was measured at 590 nm. ALP activity was calculated from a standard curve using known enzyme concentrations.^13^ The final ALP data (expressed as percentages relative to the osteogenic DMEM control – 100%) were normalized by the corresponding cell viability values (expressed as percentages relative to the DMEM control – 100%) obtained using the resazurin assay.

For Alizarin Red S staining (Sigma-Aldrich), the procedures followed the same protocol described above but extended to 13 days to allow evaluation of mineralized nodules. Images of cell cultures stained with Alizarin Red S were obtained using an inverted microscope, and staining intensity was analysed using Image J software (version 1.48; NIH, Bethesda, MA, USA). After image acquisition, 10% cetylpyridinium chloride in PBS was added to the wells and kept under agitation for 15 min to dissolve the Alizarin Red S complexes. Three 100 μL aliquots from each well were transferred to a new microplate and analysed in a spectrophotometer at a wavelength of 562 nm. The results were expressed as percentages relative to the osteogenic DMEM control

### In vivo analysis

Thirty-two 6-week-old male BALB/c mice (20 g) were housed in a controlled environment with regulated temperature (22–24ºC) and light (12-h light/dark cycle) and had free access to food and water (*ad libitum*). The study was approved by the local Ethics Committee (CEUA- 09/2020) and conducted according to the Guide for the Care and Use of Laboratory Animals of the National Institutes of Health (Bethesda, USA). The number of animals was established based on protocols from a previous study, considering an alpha error of 0.05 and a statistical power of 95% to detect a significant difference of 1 in the median scores; thus, a minimum of seven animals per group was considered necessary. To account for possible complications that could result in animal death, one additional animal was included for each group and period.^14^

Sixty-four polyethylene tubes (Abbot Lab. Do Brasil Ltda., São Paulo, Brazil) with an internal diameter of 1.0 mm, external diameter of 1.6 mm, and length of 10.0 mm, in accordance with ISO 10993-6 standard,^15^ were filled with white MTA-Angelus, PBS Cimmo HP, or Bio-C Repair Ion+; empty tubes were used as the control.

After anaesthesia, the animals had their dorsal region shaved and disinfected with a 5% iodine solution. A 2-cm incision was made in a head-to-tail orientation using a #15 blade. The skin was then reflected to create one pocket on each side of the incision. Two tubes were randomly implanted into the pockets, and the skin was sutured with 4-0 silk sutures. For postoperative analgesia, the animals received a single subcutaneous injection of dipyrone (150 mg/Kg).

At 7 and 30 days, the animals were euthanized, and the implanted tubes along with the surrounding tissues were removed and fixed in 10% buffered formalin (pH 7.0). The samples were embedded in paraffin and serially sectioned into 5 μm slices for haematoxylin-eosin or picrosirius red (PSR) staining, while 10 μm sections were prepared for von Kossa (VK) staining or left unstained for polarised light (PL) analysis.

Histological analyses were performed by a single calibrated and blinded operator using light microscopy (DM 4000 B; Leica Microsystem, Wetzlar, Germany). Tissue inflammation was scored as follows: 1. no or minimal inflammatory cells and no reaction; 2. fewer than 25 cells and a mild reaction; 3. between 25 and 125 cells and a moderate reaction; and 4. 125 or more cells and a severe reaction.^16^ Fibrous capsules were classified as thin when the < 150 μm and thick when ≥ 150 μm.^16,17^ Collagen maturation was assessed in sections stained with PSR. Greenish-yellow fibres were classified as immature and thin, whereas yellowish-red fibres were considered mature and thick.^16^After colour selection, the software automatically calculated the marked area of each collagen fibre type (Leica QWin V3, Leica Microsystems). Positive structures for VK and PL were recorded as either absent or present.^7^

### Statistical analysis

Data from cell viability, ALP activity, and mineralized nodule formation were analysed using one-way ANOVA followed by Tukey’s test for comparisons among experimental groups and Dunnett’s test for comparison with the control group (SPSS 17.0 software; SPSS Inc, Chicago USA). Data from the *in vivo* analysis were analysed using the Kruskal-Wallis test followed by Dunn’s test. A significance level of p < 0.05 was adopted for all analyses.

## Results

### In vitro analysis

**Figure 1:**
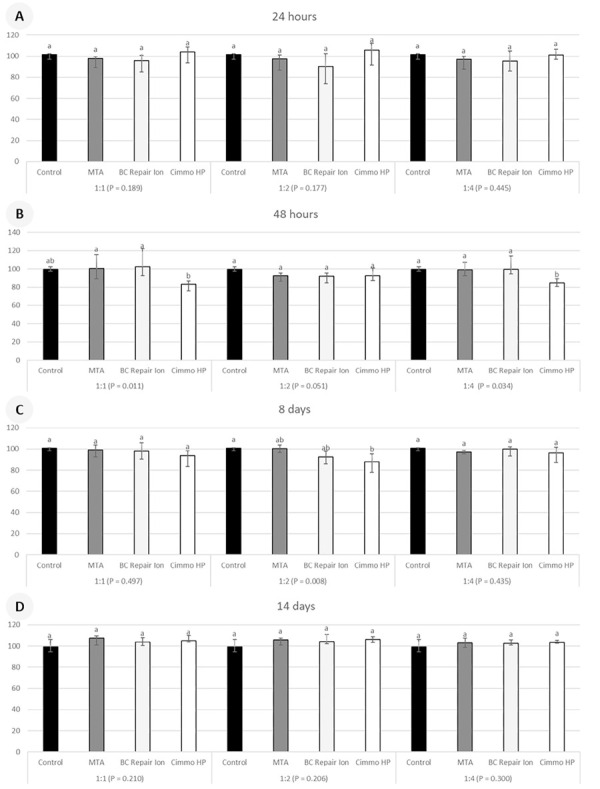
Effects of material extracts on Saos-2 cell viability after 24 h, 48 h, 8 days, and 14 days, as determined by the Alamar Blue reduction assay.

Data from the cell viability analysis are presented in Figure 1. At 24 h, all extracts of materials showed cell viability comparable to the control group (p > 0.05). At 48 h, a reduction in cell viability was observed for the 1:4 extract of PBS Cimmo HP compared with the other groups (p < 0.05). At 8 days, all extracts showed cell viability similar to the control group, except for the 1:2 extract of PBS Cimmo HP, which exhibited a significant reduction (p < 0.05). At 14 days, no differences were observed among the groups (p > 0.05).

Different letters indicate statistical differences among the groups within each extract (p < 0.05).

**Figure 2:**
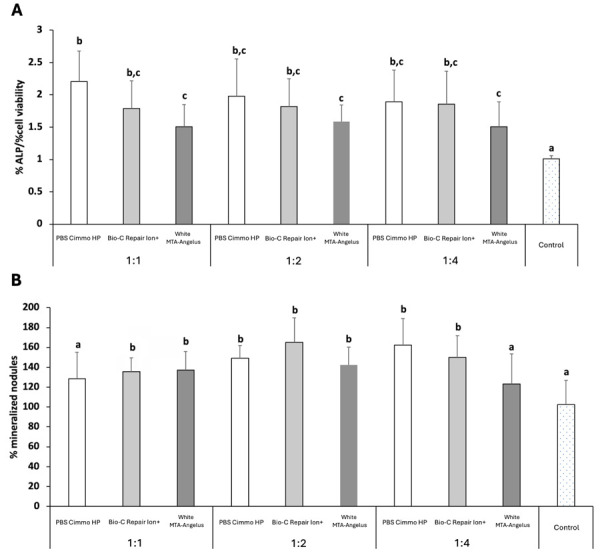
*In vitro* bioactivity analysis.

Data on ALP activity and formation of mineralization nodules are shown in Figure 2. Significant ALP activity was observed in all material groups (p < 0.05). PBS Cimmo HP exhibited significantly higher ALP activity than white MTA-Angelus in the undiluted extract, whereas no differences were observed between these materials in the 1:2 and 1:4 extracts. Bio-C Repair Ion+ did not differ from either PBS Cimmo HP or white MTA-Angelus in any dilution (p > 0.05).

(A) ALP activity after cells exposure to material extracts at 8 days. (B) Quantitative analysis of mineralized nodules after 13 days of cell exposure to material extracts. Different letters indicate statistical differences among the groups (p < 0.05).

White MTA-Angelus and Bio-C Repair Ion+ induced a significantly higher number of mineralized nodules in the undiluted extract compared with the control group (p < 0.05). All material groups showed a significant increase in mineralized nodule formation with the 1:2 extract compared with the control (p < 0.05). PBS Cimmo HP and Bio-C Repair Ion+ also showed increased mineralized nodule formation with the 1:4 extract compared with the control (p < 0.05), which was not observed for white MTA-Angelus (p > 0.05).

### In vivo analysis

**Figure 3:**
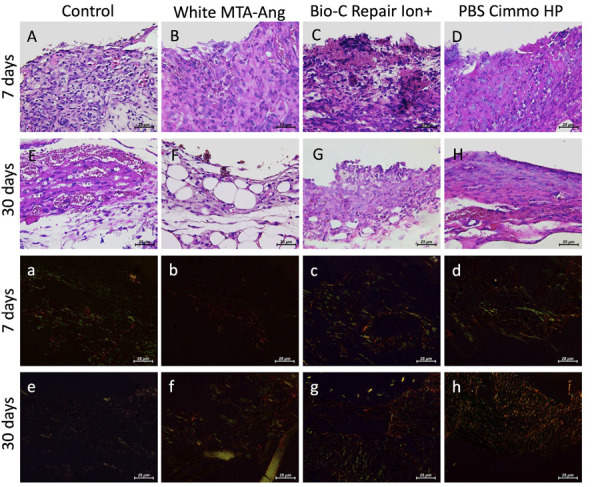
Representative images of inflammatory response and collagen maturation.

Representative images of biocompatibility and collagen maturation are presented in Figure 3, and the corresponding data are shown in Tables 1 and 2. At 7 days, control, Bio-C Repair Ion+, and white MTA-Angelus groups showed moderate inflammation, while PBS Cimmo HP exhibited moderate to severe inflammation; however, no significant differences were observed among the groups (p > 0.05). The fibrous capsule was classified as thick in all groups at this time point.

The control group at 7 and 30 days showed a thick fibrous capsule and moderate inflammatory reaction (A), with reduction in fibrous capsule thickness and mild inflammatory reaction at day 30 (H). White MTA-Angelus at 7 days showed thick fibrous capsule formation and moderate inflammatory cell infiltration (B); after 30 days (F), a reduction in fibrous capsule thickness and mild inflammatory cell infiltration were observed. Bio-C Repair Ion+ at 7 days also shows thick fibrous capsule formation and moderate inflammation (C); after 30 days (G), a thin fibrous capsule with mild inflammatory reaction was observed. After 7 days, PSR staining revealed that the (a) control and (d) PBS Cimmo HP groups exhibited a higher presence of immature collagen fibers compared with the (b) white MTA-Angelus and (c) Bio-C Repair Ion+ groups, which exhibited a significantly higher proportion of mature fibers. At 30 days, all materials showed higher proportion of mature fibers, whereas the control group displayed a relatively similar distribution of mature and immature fibers. [Hematoxylin-eosin and Picrosirius red staining, 400×]

At 30 days, most specimens from the control, Bio-C Repair Ion+, and white MTA-Angelus groups showed mild inflammation, whereas PBS Cimmo HP showed mild to moderate inflammation, with no significant differences among the groups (p > 0.05). The fibrous capsule was thin in all specimens from the control, Bio-C Repair Ion+, and white MTA-Angelus groups, and in most specimens from the PBS Cimmo HP group.

**Figure 4:**
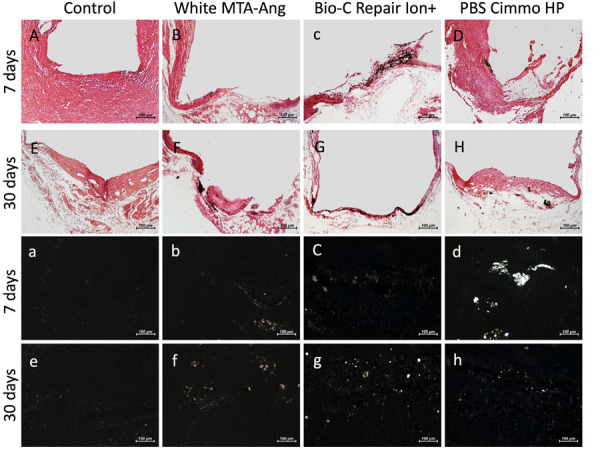
Representative images of *in vivo* bioactivity.

At 7 days, the control and PBS Cimmo HP groups showed a higher proportion of immature collagen compared with Bio-C Repair Ion+ and white MTA-Angelus, which exhibited significantly more mature fibres (p < 0.05). At 30 days, all material groups showed a predominance of mature collagen (p > 0.05), whereas the control group presented relatively similar amounts of mature and immature fibres. In both experimental periods, positive structures for VK and PL were observed in all material groups (Figure 4 and Table 1).

The control group at 7 and 30 days showed absence of dystrophic calcification (A, E) and absence of birefringent structures under polarized light (a, e). For all materials groups (B-H, b-h), in both analysis periods, positive structures for von Kossa staining and birefringent under polarized light were observed. [von Kossa staining and polarized light analysis, 100×]

**Table 1:** Inflammation scores, thickness of the fibrous capsule and *in vivo* bioactivity of the groups in each analysis period.

Period/p-value	Groups*	Scores				Median	Fibrous capsule		Bioactivity (%)	
		1	2	3	4		Thin	Thick	VK	PL
7 days/= 0.139	Control^a^	0/8	2/8	4/8	2/8	3	0/8	8/8	0	0
	White MTA-Angelus^a^	0/8	3/8	4/8	1/8	3	0/8	8/8	100	100
	Bio-C Repair Ion+^a^	0/8	0/8	6/8	2/8	3	0/8	8/8	100	100
	PBS Cimmo HP^a^	0/8	0/8	4/8	4/8	3,5	0/8	8/8	100	100
30 days/= 0.071	Control^a^	1/8	5/8	2/8	0/8	2	8/8	0/8	0	0
	White MTA-Angelus^a^	2/8	6/8	0/8	0/8	2	8/8	0/8	100	100
	Bio-C Repair Ion+^a^	0/8	6/8	2/8	0/8	2	8/8	0/8	100	100
	PBS Cimmo HP^a^	0/8	4/8	4/8	0/8	2,5	7/8	1/8	100	100

*Same letters indicate no statistical difference concerning the inflammatory response among the groups in each analysis period (p > 0.05).

**Table 2:** Percentage of mature and immature collagen fibers in each group at 7 and 30 days.

Groups*	7 days		30 days	
	Mature	Immature	Mature	Immature
Control^Aa^	37.1 ± 6.7	62.9 ± 6.7	46.6 ± 10.3	53.4 ± 10.3
White MTA-Ang^Bab^	66.9 ± 9.8	33.1 ± 9.8	63.5 ± 18.7	36.5 ± 18.7
Bio-C Repair Ion+^Bab^	71.3 ± 12.7	28.7 ± 12.7	61.9 ± 17.2	38.1 ± 17.2
PBS Cimmo HP^Ab^	40.7 ± 15.4	59.4 ± 15.4	77.6 ± 13.8	22.4 ± 13.8
p-value	< 0.001		#CAMPO!	

*Different uppercase and lowercase letters indicate significant difference between the groups regarding the maturation of collagen fibers at 7 and 30 days, respectively (p < 0.05).

## Discussion

This study evaluated the biological response of bioceramic repair materials PBS Cimmo HP and Bio-C Repair Ion+ compared with white MTA-Angelus. Most extract of materials were cytocompatible and showed *in vitro* bioactivity. *In vivo*, all materials were biocompatible and exhibited bioactivity at both 7 and 30 days. PBS Cimmo HP exhibited a greater amount of immature collagen fibres at 7 days; however, at 30 days all material groups predominantly exhibited mature collagen fibres. Therefore, the null hypothesis stating that there would be no differences among the materials regarding cytotoxicity, *in vitro* bioactivity, biocompatibility, collagen maturation, and *in vivo* bioactivity was rejected.

In this study, both *in vitro* and *in vivo* experimental designs were employed to provide a comprehensive biological evaluation of the tested materials. *In vitro* assays allowed the assessment of cellular responses under controlled conditions, whereas the *in vivo* subcutaneous implantation model enabled the evaluation of tissue responses such as inflammation, fibrous capsule formation, collagen maturation, and mineral-related deposition within a physiological environment.^7,14,18^ The combination of these approaches supports a broader interpretation of the biological behavior of the materials.

The cytotoxicity of the materials was evaluated using osteoblast-like cells (Saos-2). Selecting an appropriate cell line is crucial for the biological analysis of the dental materials.^19^Saos-2 cells are commonly used to evaluate the cytocompatibility and bioactivity of new dental materials.^20-24^ Cells of the osteoblastic lineage should be prioritized when evaluating materials designed for dental tissues, as these materials will be placed in contact with periapical tissues or pulp tissue and its odontoblasts. However, it should be recognized that, as a transformed cell line, Saos-2 cells may not fully represent primary dental pulp or mesenchymal cells.^19^

Three different dilutions of the repair materials extracts were evaluated over a 14-day period to assess the diffusion of compounds into tissues. Most materials showed cell viability comparable to the control group, excepted for the 1:4 extract of PBS Cimmo HP at 48 h, which showed reduced cell viability. A previous study reported reduced cell viability for Bio-C Repair Ion+ in NIH3T3 fibroblasts at 24 h and 7 days compared with the control group, although it presented lower cytotoxicity than white MTA-Angelus.^10^ Although a reduction in cell viability was observed in the study of Klein-Junior et al.,^10^ Bio-C Repair Ion+ demonstrated satisfactory cytocompatibility compared with other repair materials, similarly to the viability results observed in the present study.

The toxic effects of undiluted PBS Cimmo HP have been reported in human periodontal ligament stem cells (hPDLSCs), particularly up to 72 h, using the MTT assay.^5,6^ Although Santiago et al.^23^ reported ideal viability rates using undiluted PBS Cimmo HP in Saos-2 cells, they observed low migration rates after 24–48 h of exposure. Some components of calcium silicate cements may affect the contractile system and the expression of adhesion molecules, thereby impairing mechanical properties.^23^

The mineralization capacity of the new repair materials was evaluated by ALP activity and Alizarin Red staining, which detects mineralized nodules-specifically calcium-rich deposits-produced by osteoblast-like cells, representing key indicators of cell differentiation after biomaterial contact.^19^ ALP is expressed during early osteoblastic maturation and is responsible for dentin matrix mineralization.^19,22^ The ALP activity results demonstrated the all materials induced mineralization in osteoblast-like cells within 8 days, particularlyundiluted PBS Cimmo HP, which showed higher ALP activity compared with white MTA-Angelus. Significant mineralized nodule formation was also observed for all tested materials.

These findings are consistent with a previous study in which undiluted PBS Cimmo HP also demonstrated mineralization potential after 5 days.^23^ However, Pedrosa et al.^6^ found no significant ALP activity after 7 days of exposure to a 1:16 dilution of PBS Cimmo HP in untreated hPDLSCs. These divergent results may be related to the different cell types used and the high dilution of the PBS Cimmo HP extract (1:16) applied by the authors in their *in vitro* model, as undiluted PBS Cimmo HP demonstrated superior mineralization compared with the other tested materials and dilutions in the present study. Regarding the satisfactory performance of Bio-C Repair Ion+, the metal ions incorporated into its formulation are proposed by the manufacturer to enhance its bioactive potential, which may have contributed to its osteogenic differentiation.^10,25^

Regarding white MTA-Angelus, cytotoxicity results reported in different studies are controversial. Some studies have shown that white MTA-Angelus exhibits cytocompatibility similar to or even higher than that of the control in most extracts,^7,9,24,26^ whereas others have reported cytotoxic effects.^9,24^ These differences may be explained by several factors, such as the type of cells used, variations in extract preparation and concentration, and differences in the analysis time points. Overall, however, MTA is generally regarded as a cytocompatible material, which has also been confirmed in *in vivo* studies.^27^

The association of these *in vitro* tests with histological analysis in animals provides preliminary safety data and helps predict clinical responses.^19,27^ In this study, the early cellular responses observed *in vitro*, including cell viability and ALP activity, were consistent with the tissue responses observed *in vivo*, such as the initial inflammatory reaction, fibrous capsule formation, collagen maturation, and mineral-related deposition.

The implantation of polyethylene tubes containing the materials into the subcutaneous tissue of animals is a standardized method according to ISO 10993-6.^15^ This model, supported by clinical studies, allows the evaluation of tissue inflammation and fibrous capsule thickness as indicators of biocompatibility. Furthermore, this approach enables the assessment of bioactivity and collagen maturation in connective tissue.^7,17^ In this context, von Kossa (VK) staining identifies phosphate-containing mineral deposits, whereas polarized light (PL) microscopy reveals birefringent crystalline structures, contributing to the interpretation of the biological behavior of the tested materials.^18^

MTA has been proven to be effective due to its high biocompatibility and is widely used in various endodontic applications.^2^ The precursor of Bio-C Repair Ion+, Bio-C Repair, showed results similar to those of MTA in a previous *in vivo* study.^7^ The results of the *in vitro* and histological evaluations in the present study corroborate previous *in vitro* findings that reported mild to moderate cytotoxicity for Bio-C Repair Ion+ at 24 h and 7 days, respectively.^10^ The metal ions recently incorporated into its formulation did not negatively influence the biocompatibility of the material. However, PBS Cimmo HP had not previously been evaluated in an animal model.

Bio-C Repair Ion+, similarly to its precursor, induced biomineralization. Histologically, it can be inferred that the compositional modification did not impair its biological properties, including its biomineralization potential. As this was the first study to evaluate *in vivo* bioactivity of PBS Cimmo HP, although biomineralization induction was observed-even if at a later stage-this still represents an initial investigation, and further studies, particularly using additional animal models, are necessary. These findings are consistent when the *in vitro* bioactivity results observed in the present study, confirming the ability of tricalcium silicate cements to stimulate mineralization*.* The bioactivity of white MTA-Angelus was also confirmed once again.

Considering the results, tricalcium silicate-based cements increase the environmental pH due to their high release of OH- ions, which appears to provide a favourable environment for osteoblastic cell lines,^21,23^ in addition to enhancing ALP activity and contributing to antimicrobial properties.^22,24^ Moreover, the calcium ions released from calcium hydroxide leached from hydrated materials play a crucial role in the differentiation and proliferation of hard tissue-forming cells.^23,28^

Picrosirius red staining allows differentiation of collagen types under polarized light. A yellow-green colour suggests immature and thin collagen fibres, whereas an orange-red colour indicates mature and thick fibres.^26^ This is the first study to assess collagen maturation in tissues in contact with white MTA-Angelus and the new materials. The precursor of Bio-C Repair Ion+, Bio-C Repair, had also not previously been evaluated for collagen maturation. Therefore, it is not possible to directly compare these results with previous studies on repair materials.

This study presents some limitations that should be acknowledged. The use of a single osteoblast-like cell line for the *in vitro* analyses may not fully represent the biological responses of other cell types involved in dental tissue repair. In addition, the evaluation periods of 7 and 30 days, although adequate to assess early and intermediate tissue responses, do not capture long-term outcomes. Finally, although the subcutaneous implantation model is well established for biocompatibility assessment, it does not fully reproduce the complex environment of dental or bone tissues. Therefore, the findings should be interpreted within these limitations, and future studies are warranted to further investigate the biological behaviour of these materials under different experimental conditions.

Despite the need for prospective clinical studies to confirm biological performance, the present findings contribute to the understanding of the biocompatibility and bioactivity of these materials when in contact with living cells and tissues. Bio-C Repair Ion+ and PBS Cimmo HP showed biocompatibility comparable to white MTA-Angelus. Both materials also induced bioactivity, although this biological response appeared to be delayed for PBS Cimmo HP. Greater collagen maturation was observed at 30 days, which may indicate a process of tissue repair and fibrosis organization. The maturation pattern observed alongside reduced tissue inflammation is a positive finding, in addition to the presence of structures positive for mineralization. Although subcutaneous tissue is not a mineralizing environment, these findings may indicate a favourable repair response in connective tissue. Furthermore, the positive bioactivity observed indicates that, when in contact with bone tissue, these materials my stimulate hard tissue formation, which should be further investigated. Overall, Bio-C Repair Ion+ and PBS Cimmo HP influenced collagen maturation similarly to white MTA-Angelus.

## Conclusion

PBS Cimmo HP and Bio-C Repair Ion+ were cytocompatible and biocompatible, showed bioactivity, and promoted collagen maturation similar to that observed with white MTA-Angelus.

## Data Availability

The datasets generated during and/or analyzed during the current study are available from the corresponding author on reasonable request.
